# Street Harassment Interpretations: An Exploration of the Intersection of Gender and Race/Ethnicity, and Mediator Variables

**DOI:** 10.1177/10778012221094067

**Published:** 2022-08-09

**Authors:** Jennifer Herrera, Bill McCarthy

**Affiliations:** 1 8789 University of California, Davis, CA, USA; 2 School of Criminal Justice, 140556 Rutgers University, Newark, NJ, USA

**Keywords:** street harassment interpretations, gender, race/ethnicity, sexual harassment myths

## Abstract

How does the intersection of gender and race/ethnicity influence street harassment interpretations? What roles do attitudes and past experience play in these relationships? We examined these questions through an exploratory study of 163 Californian respondents and four hypothetical scenarios: being told to smile, being called “sexy,” hearing kissing noises, and being followed. Our findings revealed Black, Latina, and White women were more critical of these behaviors than men in their race/ethnic group. Women across all race/ethnicities interpreted the scenarios similarly with minor nuances. Street harassment views were strongly associated with prior experiences instigating street harassment and support for harassment myths.

## Introduction

In 2014, the anti-street harassment organization Hollaback! (https://www.ihollaback.org/) commissioned a video of a White woman, Shoshana Roberts, walking for 10 hours in New York City (https://www.youtube.com/watch?v=b1XGPvbWn0A). The camera recorded her interactions with men as she experienced over 100 catcalls, whistles, leers, and being followed. The video sparked an international conversation about the definitions and the behaviors that constitute street harassment along with the racial bias that overrepresented men of color doing the harassment. An analysis of 1,000 YouTube comments on the video offered a window into the interpretations discussion: most comments characterized the actions as compliments made by well-intentioned men; few labeled them as invasive or harassment (Bailey, 2017a).

Street harassment is a damaging “rite of passage” for women (Kearl, 2015, p. 29), a reminder of their vulnerability to sexual assaults (Bowman, 1993; Davis, 1994; Fogg-Davis, 2006; Kissling, 1991) and an example of sexual terrorism that increases fear in public places (Macmillan et al., 2000; Vera-Gray & Fileborn, 2018). Research has documented the negative psychological consequences among women: a heightened fear of rape (Fairchild & Rudman, 2008), posttraumatic stress disorder symptoms (Carretta & Szymanski, 2020), poor quality of sleep (DelGreco & Christensen, 2020), as well as the internalization of negative comments made about one's body (Davidson et al., 2015). Women experience street harassment at alarming rates (Gardner, 1995; Kearl, 2014, 2015, 2018; Nielsen, 2000). In a nationally representative United States online study of 2,000 respondents, 77% of women reported experiencing forms of street harassment, compared to 34% of men (Kearl, 2018). Transgender women and men, nonbinary people, and those with disabilities experience even higher rates (DC Office of Human Rights, 2020; Kearl, 2014, 2018). Victims experience this harm in ways that interweave with racism, anti-blackness, classism, ableism, transphobia, and xenophobia.

Sparse studies on interpretations report women mainly interpret street harassment as rude, offensive, objectifying, and hostile (Gardner, 1995; Kearl, 2014, 2015, 2018; Kissling, 1991; Nielsen, 2000). Yet, assessments are complex and some women view some forms of street harassment as complimentary, flattering, and/or harmless (Fairchild, 2010; Gardner, 1995; Kissling, 1991; Kissling & Kramarae, 1991). Meanwhile, men disproportionately perceive street harassment as romantic, fun, or inconsequential encounters (Benard & Schlaffer, 1984; Gardner, 1995; Kissling, 1991) and claim that their acts are predominately motivated by affection (DelGreco et al., 2020). While the street harassment research seemingly presents a gender divide in views, it has mostly abstracted gender *and* race, therefore treating them as separate categories, along with other identities and statuses. As well, an overwhelming majority of studies have focused on the experiences of heterosexual, White, cis-gender (those that align with the sex given at birth) middle-class, women—the paradigmatic victim. The aim of this exploratory study is to use an intersectional approach to analyze how Black, Latinx, and White women and men view street harassment interactions. The analysis compares views among women and men in the same race/ethnic group along with women's interpretations in comparison to each other. Additionally, we examine whether support for sexual harassment myths and experiences instigating street harassment intervene between race/ethnicity, gender, and street harassment views. Our study included both victims and perpetrators of street harassment.

## Definition

Several terms are attributed to the behaviors we study: offensive public speech (Nielsen, 2000), men's stranger intrusions on women in public space (Vera-Gray 2016), street remarks (Bailey, 2016, 2017a, 2017b; Kissling & Kramarae, 1991), stranger harassment (Fairchild & Rudman, 2008; Wesselmann & Kelly, 2010) and street harassment (Bowman, 1993; Davis, 1994; DelGreco et al., 2020, di Leonardo, 1981; Fogg-Davis, 2006; Hutson & Krueger, 2019; Kearl, 2014, 2015, 2018; Kissling, 1991; McNeil 2014; Whatley, 2018) along with others. We use the term “street harassment.” First, it is commonly used outside of academic circles in the United States and in prominent campaigns, such as Tatyana Fazlalizadeh's “Stop Telling Women to Smile” artwork. Hollaback's trainings also use “street harassment.” Media coverage and policies—such as The Street Harassment Prevention Act in 2018—have also helped popularize this term. Secondly, there is a growing understanding that street harassment is distinct from workplace sexual harassment in part because the latter provides more legal protections. Hutson and Krueger's (2019, p. 770) definition touches on these elements:

Street harassment constitutes unwanted attention in public, which psychologically, emotionally, and/or physically impinges on the target's well being…[it] is an intrusion, often by a person unknown to the target, which may take a variety of forms, ranging from remarks on physical appearance to sexual touch to brutal physical assaults.

While some definitions center on men's harassment toward women (e.g., Vera-Gray, 2016), Hutson and Krueger's (2019) expansive definition captures harassment toward people of all genders. Commonly cited examples of street harassment include ogling, catcalling, honking, and groping; some scholars contend that it also includes rape (Bowman, 1993).

## Street Harassment Interpretations and Gender

Studies have reported women and men construct and experience this harm in ways that reflect socially constructed gender roles and power hierarchies that are reproduced in public spaces (Ridgeway & Correll, 2004; West & Fenstermaker, 1995). In one of the earliest large-sample studies on street harassment—and one of the few studies to investigate perceptions—Gardner (1995) interviewed 506 women and men in Indianapolis, Indiana. Her analysis posits two dominant street harassment perspectives: the politicized feminist and romanticized traditionalist.

The politicized feminist perspective frames street harassment as a consequence of inequalities between women and men and part of a continuum of discrimination women face at school, the workplace, and in public spaces. The male gaze, leers, winks, and other forms of street harassment function as reminders of women's lower statuses and the social acceptability of men's evaluation of them as sexual objects. Most women in Gardner's (1995) study supported the politicized feminist view. Other studies echo a similar trend: most women interpret street harassment as offensive, threatening, or invasive (Davis, 1994; DelGreco et al., 2020; di Leonardo, 1981; Kissling, 1991; Nielsen, 2000).

According to the romanticized traditionalist perspective, street harassment is flattering, trivial, and/or an invitation women welcome by their clothing or physical appearance. Grounded in heteronormativity, the view assumes women desire to have their attractiveness affirmed by heterosexual men with the hope “something significant could eventuate” between them (Gardner, 1995, p. 164). This heteronormativity—the upholding of heterosexuality as the norm and superior form of sexuality—is a foundational power structure perpetuated and sustained through social and cultural institutions (Robinson, 2016). Most men in Gardner's (1995) study upheld the romanticized traditionalist view. Some women in Gardner's (1995) study also described certain street interactions as flattering, emphasizing that ones with younger and attractive men were more acceptable (see also Fairchild, 2010; Kissling & Kramarae, 1991).

Men's views of street harassment as complimentary, flattering, inoffensive, or as rewards for physical attractiveness have also been noted in other research. A study of 60 men who harassed women in public places documented their many justifications (Benard & Schlaffer, 1984): it is a “harmless fun,” alleviates boredom, and facilitates bonding and friendship among other men (see also Bailey, 2016; Wesselmann & Kelly, 2010). Similarly, college men argued women actively seek men's attention in public places and enjoy these street encounters (Fairchild, 2010). Although less commonly studied, research has reported that race/ethnicity are not strongly related to perpetuating harassment. Gardner (1995) noted most of the men she interviewed engaged in street harassment; it is, she concluded: “practiced by men of all types, races, and classes” (p. 111; see also Davis, 1994; Fogg-Davis, 2006; Kissling, 1991).

## Intersectionality

The street harassment discourse has primarily drawn upon the experiences of White cis-gender middle-class women. It has treated race/ethnicity as a secondary, additive axis. But, as Davis (1994) argued, no sole categorization is primary in understanding street harassment. An intersectional analysis (Collins, 2015; Crenshaw, 1991) is needed, especially one that situates experiences within multiple interlocking systems of power (Fileborn, 2018; Fileborn & Vera-Gray, 2017; Vera-Gray & Fileborn, 2018), particularly, in this case, gender and race/ethnicity. Grounded in Black feminist thought, intersectionality breaks from the single-axis framework (Crenshaw, 1991) and highlights a matrix of domination where various attributes, including gender, race, class, and sexual orientation—and others—operate in concert to construct social inequalities (Collins, 2015). An intersectional analysis examines problems of sameness and difference in relation to power (Cho et al., 2013). We build on these insights and de-essentialize street harassment as a solely gender-based phenomenon by systematically analyzing how both race/ethnicity *and* gender inform understandings of street harassment.

## Street Harassment, Gender, and Race/Ethnicity

The street harassment literature has primarily focused on theoretical arguments when considering the intersection of gender and race/ethnicity. Some authors theorize Black people hold shared sentiments about and tolerance for sexual and street harassment because of “Black cultural scripts” and social norms. For instance, Kochman (1981, p. 84), posited attempting to “pick-up” women through sexual advancements “does not violate [B]lack cultural norms” (also see Duneier, 1999). In her study, Gardner (1995) found that Black women provided a romanticized interpretation of street harassment at a higher rate than White women. Black women interviewed by Welsh et al. (2006) said they were less likely than other women to interpret sexual behaviors as sexual harassment and they maintained they were better equipped to handle these interactions because of their experiences with racial discrimination.

Given the historical and current experiences with law enforcement and the state, Black women may downplay the interactions in part because they have to navigate and contest with racial loyalty with Black men. As Bent-Goodley (2001, p. 323) explains, Black women “may withstand abuse and make a conscious self-sacrifice for what she perceives as the greater good of the community but to her own physical, psychological, and spiritual detriment.” Black women are disproportionately assaulted, arrested, murdered, as well as sexually assaulted by the police (Crenshaw et al., 2015), thereby limiting their turning to the legal system for support (Jacobs, 2017). They must also contend with racist stereotypes about being angry or hypersexualized that limit how they respond to street harassment (Vera-Gray & Fileborn, 2018).

Other scholars assert that Black women recognize the true harms of street harassment. According to Fogg-Davis (2006), many Black women acknowledge the roots of street harassment in Black patriarchy and its use by Black men to intimidate and control them. In a Harlem ethnography, Jackson (2001, pp. 148–149) noted that Black women described street remarks as “disrespectful” and “frustrating.” Of the few studies that have systematically examined how *both* race and gender, findings report Black women experience street harassment more frequently than White women (Kearl, 2014; Nielsen, 2000; Watson et al., 2015) and that they are often furious when harassed by Black men (Kearl, 2015).

Within the Latinx community, cultural norms grounded in heteropatriarchal beliefs may also influence street harassment interpretations. Achugar (2001) noted that Spanish-speaking communities share a historical bond reflected in linguistic practices. She argued *piropos*—comments men make in public to women whom they do not know—are demonstrations of *machismo and marianismo ideology*. The former includes beliefs and values about masculinity and dominance over women, while the latter involves gender norms for women influenced by beliefs about the Virgin Mary. According to Achugar (2001), Spanish-speaking cultures have traditionally interpreted *piropos* as compliments or praise (see also Bailey, 2017a, 2017b; Moya-Garófano, et al., 2018).

Other scholars have suggested the interpretation of *piropos* is not uniformly shared by Latinas. For example, Achugar (2001) found that, while older women were more likely to see *piropos* as polite, younger women more often viewed them as harassing. The Latinas Kearl (2015) spoke with routinely characterized *piropos* as disgusting and disrespectful.

## Mediators: Sexual Harassment Myths and Prior Experience Instigating Street Harassment

Most research on street harassment has overlooked the role of factors that may intervene between the intersection of race/ethnicity, gender, and street harassment views. We considered two potential mediators. The first, support for sexual harassment myths, is grounded in research on sexual harassment myths. According to Lonsway et al. (2008), sexual harassment myths are beliefs that women exaggerate its extent, along with its intensity, and bring victimization upon themselves. They are associated with justifications for sexual advances and denials of the severity of sexual harassment (Rotundo et al., 2001). Street harassment is a form of sexual harassment (DelGreco & Christensen, 2020; DelGreco et al., 2020; Fogg-Davis, 2006) and, as Gardner (1995) detailed, beliefs about the acceptability of harassment are common, especially among men and women who embrace a romanticized traditionalist perspective.

Our second intervening variable is prior experience engaging in street harassment. Men are overwhelmingly the instigators of street harassment (Benard & Schlaffer, 1984; DelGreco et al., 2020; Gardner, 1995) and research finds that prior engagement in street harassment is associated with their predictions of participating in it in the future (Wesselmann & Kelly, 2010). Yet, research has also documented that between 20% (Kearl, 2014, p. 7) and 30% (Kearl 2018, p. 8) of men reported their harasser was a woman (or perceived them as women).

As our review demonstrates, women of all racial/ethnic groups are targets of street harassment, whereas men are usually the perpetrators. Few studies have, however, systematically examined connections between street harassment interpretations and the intersection of race/ethnicity and gender, or the mediating role of street harassment myths and experiences engaging in the behaviors. We examined these considerations in the study described below.

## Data, Measures, and Analysis

We collected data in a 2017 Institutional Review Board (IRB)-approved exploratory study. Our study builds upon a small IRB-approved pilot study conducted by the first author which was influenced by their experiences with street harassment. We trained four bilingual (English and Spanish), Latinx, undergraduate students to help collect our data: three identified as women and one identified as genderqueer. Both authors also collected data and supervised the data collection. We gathered data between April and October, in four parks and markets in three California cities: Oakland, Richmond, and Sacramento. We chose these locations accessible to pedestrians because they are racially and educationally diverse. We administered surveys for an average of 3 hours a day, mostly during the late morning and afternoon, on 22 occasions total.^1^

We used a variation of systematic random sampling to invite potential respondents. Under the supervision of one of the authors, data collectors invited every tenth person that walked by to participate in a 10- to 15-min study. In times of slow foot traffic, the data collectors invited every fifth person. When a respondent agreed to take the survey, the data collectors started a new count for selecting the next potential participant after they completed the survey. If the invited participant declined to participate, the data collectors started the count again.

Data collectors identified themselves as university researchers conducting a study on interactions in public places. They informed potential participants they were randomly chosen and would be included in a lottery for a $25 gift card, regardless of whether or not they participated in the study. Data collectors asked participants about their age to ensure they were 19 years or older. Respondents used an electronic tablet to complete an anonymous 32-question self-report survey in English or Spanish. The survey was available in different languages as research documents language accessibility and the translation of surveys into different languages increases response rates among immigrants (Moradi et al., 2010). As part of the protocol, data collectors asked the respondents their preferred language for the survey. We collected data from 194 participants.^2^

## Measures

The study has four dependent variables, which reflect interpretations of four hypothetical scenarios. We used hypothetical scenarios so that we could include participants with a range of exposure and experiences, from none to extensive, and to include perpetuators and targets of street harassment. We prefaced each scenario by reminding respondents to answer questions about each scenario, even if they had not experienced the behavior described. Due to time constraints, our scenarios were limited to gender binary interactions involving a “stranger of the opposite gender.” We had women think about men as the perpetuator, and vice versa, as it creates a constant in our study so that the participants were not thinking about same-gender harassment. Our design follows the lead of other important research that also includes women and men in their sample (see Gardner, 1995; Kearl, 2018). A small set of respondents who identified as transgender or nonbinary, along with other genders (*n*  =  5), completed the survey and shared how they interpreted “a stranger of the opposite gender,” but we could not include them in this analysis because of the small sample size.

The survey included a total of four scenarios including verbal, nonverbal, and physical interactions. The first question asked: “A stranger from the opposite gender asks ‘Can I get a smile?’ and then says ‘Smile for me’ while you are walking down the street” (smile scenario). The second scenario asked: “A stranger of the opposite gender says ‘Hey Sexy!’ or something similar while you are walking down the street” (hey sexy scenario). The third scenario involved a nonverbal, suggestive interaction: “A stranger of the opposite gender makes kissing noises to you as you walk by while you are walking down the street” (kissing noises scenario). The final scenario included a physical component: “A stranger of the opposite gender says ‘Hey baby!’ to you and proceeds to follow you down the street” (being followed scenario). Following each scenario, there were five questions presented. The first three asked to what extent they agreed or disagreed (1  =  *strongly agree* … 7  =  *strongly disagree*) the actions were: (1) compliments, (2) polite, or (3) harassment (reverse coded). Afterward, we asked, “How often has a stranger said remarks like this *to you* in public places?” and “How often have *you* said remarks like this to a stranger when walking down the street?” Both questions had five response categories (0  =  *Never*, 1  =  *Rarely*, 2  =  *Occasionally*, 3  =  *A lot*, 4  =  *Daily*).

We considered two ways to create scales from responses. The first approach used the *content of the scenarios* to make scales for each behavior (e.g., smile scenario scale). The second approach treated each *view* as a scale (e.g., polite scale). Goodness-of-fit statistics from structural equation models showed the first approach was superior to the second (Bayesian information criterion [BIC]  =  6896.86 vs. 7063.85, df  =  36) so we used the answers of each scenario to create scales. Ordinal alpha (Gadermann et al., 2012) for the scales are as follows: smile scenario (0.79), hey sexy scenario (0.85), kissing noises scenario (0.87), and being followed scenario (0.85).

We measured our key independent variables with respondents’ gender and racial/ethnic identity responses. We followed Crenshaw's (1991) recommendation to examine race/ethnicity and gender together rather than separately and created six categorical variables: White women (comparison group, *n*  =  32), White men (*n*  =  24), Latinas (*n*  =  24), Latinos (*n*  =  30), Black women (*n*  =  24), and Black men (*n*  =  29). Following a pattern established by other scholars (Western et al., 2015), we coded anyone who selected a multiracial identity (*n*  =  9) as Black or Latino/a if that was part of their identity; no one in our sample identified as Black and Latino/a. Due to a small sample size, we could not include respondents who identified as Asian (*n*  =  9).

We measured support for street sexual harassment myths (one of our mediator variables) with a three-item scale (ordinal alpha  =  .61) adapted from the Illinois Sexual Harassment Myth Acceptance (ISHMA) Scale (Lonsway et al., 2008). The ISHMA scale focuses on workplace sexual harassment, so we replaced the word “work” with “public spaces.” These questions were located in earlier sections of the survey, separate from the hypothetical scenarios: (1) “Most women are flattered when they get sexual attention from men in public spaces,” (2) “It's inevitable that men will ‘hit on’ women in public spaces,” and (3) “Women can usually stop unwanted sexual attention by simply telling the man that his attention is not appreciated.” The three questions had seven Likert-scale responses (1  =  *strongly disagree* … 7  =  *strongly agree*).

Other identities and statuses may also influence interpretations. To capture this, we included ordinal-measure controls for four demographic attributes: sexual orientation, age, education, and income. We used a dichotomous measure of sexual orientation (0  =  bisexual, gay/lesbian, or other; 1  =  heterosexual/“straight”). Our age variable had six categories (19–24; 25–30; 31–35; 36–40; 41–45; 46+), education had eight (no schooling completed; some high school, no diploma; trade, technical, or vocational training; high school graduate, diploma or the equivalent (e.g. General Educational Development); some college credit, no degree; associate degree; bachelor's degree; master's, professional or doctorate degree), as did income ($0–$15,000; $15,001–$30,000; $30,001–$45,000; $45,001–$60,000; $60,001–$75,000; $75,001–$90,000; $90,001–$105,000; >$105,001). Given the high levels of harassment reported in earlier research, particularly from women (e.g., Kearl, 2014, 2018), we included an ordinal measure of the frequency of having been the target of each behavior (0  =  never, 1  =  rarely, 2  =  occasionally, 3 =  a lot, 4  =  daily).

## Analysis

Our analysis began with descriptive statistics of differences across gender and race/ethnicity. We then used ordinary least squares regression to analyze responses to the four scenarios. There was a high item response rate for most measures, but we used imputed data (30 multiple imputed datasets) to maximize power. We did not impute dependent variables; therefore, our analytic sample is the 163 respondents who answered questions on *all four scenarios*. We estimated two models for each dependent variable. First, a base model that included our gender–race/ethnicity and control variables, and second, a full model that added support for myths about sexual harassment and prior experiences instigating it. We used a Z test (Clogg et al., 1995) to assess if coefficients from the two models were significantly different from each other.

## Results

### Gender and Race/Ethnicity Mean Differences

Descriptive data in Table 1 indicate that Black, Latina, and White women interpreted all of the four scenarios as more impolite, uncomplimentary, and harassing in comparison to men in their respective race/ethnic group. One-way analysis of variance tests of mean differences indicate the differences are statistically significant. Of the surveyed race/ethnic groups, *White women rated all the scenarios most critically,* followed by White men, Latinas, Black women, Black men, and Latinos. White women, Latinas, and Black women all had significantly less average support for street harassment myths in comparison to men in their race/ethnic group. Men from each group reported higher levels of initiating the four behaviors than women, whereas women in each group reported higher levels of having been the target of each behavior.

**Table 1.** Descriptive Statistics, One-Way Analysis of Variance (ANOVA) Test of Mean Differences by Gender Within Racial/Ethnic Groups.^a^

	White women		White men		Latinas		Latinos		Black women		Black men	
Dependent variables	X	*SD*	X	*SD*	X	*SD*	X	*SD*	X	*SD*	X	*SD*
Smile scenario***	5.84	1.03	5.22	1.13	5.12	1.45	3.79	1.23	4.51	1.33	3.88	1.50
Hey sexy scenario***	6.06	1.04	5.21	1.17	5.44	1.15	4.28	1.61	4.83	1.52	4.24	1.32
Kissing noises***	6.46	0.89	5.96	0.91	5.64	1.32	4.64	1.65	6.01	1.24	5.15	1.35
Being followed***	6.66	0.71	5.97	1.00	5.70	1.11	4.79	1.51	6.09	1.00	5.55	1.13
Mediator variables												
Sexual harassment myth support***	3.11	0.93	3.65	1.22	3.91	1.46	4.94	1.25	4.28	1.53	4.60	1.27
Prior initiator, “Can I get a smile?"***	0.03	0.18	0.42	0.72	0.44	0.99	0.86	0.79	0.78	1.17	0.86	0.99
Prior initiator, “Hey sexy!"*	0.00	0.00	0.29	0.55	0.30	0.63	0.63	0.93	0.44	0.84	0.48	0.79
Prior initiator, kissing noises*	0.00	0.00	0.25	0.85	0.26	0.69	0.53	0.73	0.22	0.52	0.31	0.54
Prior initiator, being followed!*	0.00	0.00	0.29	0.86	0.13	0.46	0.53	0.68	0.30	0.76	0.28	0.59
Control variables												
Age	3.88	1.79	3.96	1.83	3.22	2.07	4.03	1.71	4.30	1.69	4.14	1.94
Sexual orientation (1 = heterosexual)	0.74	0.45	0.96	0.20	0.87	0.34	0.87	0.35	0.86	0.35	0.97	0.19
Education***	7.42	0.89	6.83	1.37	4.55	2.13	4.57	2.29	6.65	1.70	6.24	1.64
Income***	5.81	2.59	6.13	1.99	2.95	2.13	4.15	2.63	4.55	2.67	4.14	2.14
Prior target, “Can I get a smile?"***	2.38	0.83	0.83	0.96	2.32	0.75	1.33	0.96	2.39	0.84	1.18	0.91
Prior target, “Hey sexy!"***	2.06	0.98	0.83	0.92	2.50	0.63	0.95	0.65	2.00	0.80	0.82	0.86
Prior target, kissing noises***	1.63	0.75	0.50	0.93	2.06	0.54	1.10	1.07	1.65	0.78	0.79	0.92
Prior target, being followed***	1.52	0.93	0.71	1.08	1.80	0.68	1.00	0.84	1.61	0.84	0.52	0.69

a Based on raw data.

***p ≤ .001, **p ≤ .01, *p ≤ .05 (two-tailed).

### Multivariable Results

Table 2 displays results for gender–race/ethnicity groups and control variables (age, sexual orientation, education, income, and prior experiences being targeted) for each scenario. In this table, White women were the comparison group. None of the coefficients for Latinas were significant, suggesting their perceptions did not differ substantially from White women's views. The coefficients for Black women in the kissing noises and being followed scenarios were also not significant, indicating that Black and White women had similar interpretations. Yet, Black women marked more tolerant views of the smile and hey sexy scenario in comparison to White women. Significant negative coefficients for men from all groups indicated they saw the four behaviors as more polite, complimentary, and nonharassing in comparison to White women.

**Table 2.** Ordinary Linear Squares (OLS) Regression, Street Harassment (*n*  =  163).

Variables	Smile^a^	Hey sexy	Kissing noises	Being followed
White men	−0.76*	−1.29***	−0.65*	−0.74**
	(0.37)	(0.38)	(0.29)	(0.23)
Latinas	−0.56	−0.18	−0.31	−0.32
	(0.41)	(0.38)	(0.36)	(0.32)
Latinos	−2.00***	−2.01***	−1.54***	−1.48***
	(0.39)	(0.41)	(0.37)	(0.31)
Black women	−1.17***	−1.08**	−0.24	−0.36
	(0.33)	(0.37)	(0.30)	(0.23)
Black men	−1.89***	−2.11***	−1.24***	−1.03***
	(0.39)	(0.37)	(0.33)	(0.28)
Age	−0.07	−0.08	−0.05	0.05
	(0.06)	(0.06)	(0.05)	(0.05)
Sexual orientation	−0.20	−0.36	−0.34	−0.41
	(0.28)	(0.28)	(0.25)	(0.21)
Education	−0.01	0.06	0.11	0.14*
	(0.07)	(0.06)	(0.06)	(0.06)
Income	0.08	0.04	0.03	0.04
	(0.05)	(0.05)	(0.05)	(0.04)
Prior target	−0.11	−0.45**	−0.26*	−0.23*
(Scenario specific)	(0.12)	(0.14)	(0.12)	(0.09)
Constant	5.94***	6.53***	5.97***	5.46***
	(0.69)	(0.63)	(0.58)	(0.49)

a Unstandardized regression coefficients; robust standard errors in parentheses.

***p < .001, **p < .01, *p < .05 (two-tailed).

There are also some notable patterns for the control variables in our model. Table 2 shows scenario interpretations were generally not related to our other demographic variables (i.e., sexual orientation, age, education, and income). Table 2 also indicates that prior victimization had significant negative associations with street harassment views for three of four scenarios (smile was the exception). This suggests that the higher the frequency of victimization, the greater the acceptance of the behaviors. We explore the reason for this counter-intuitive association with additional analyses (not shown, available on request) that focused separately on each gender. In these gender-specific analyses, men's experiences being targeted were significantly and negatively related to their views about all four behaviors: men who had been the target were less likely to see the behaviors as harassment, impolite, or uncomplimentary. In contrast, for women, previous experiences being targeted were not significantly associated with views about the scenarios. In sum, men infrequently experienced the scenario behaviors (see Table 1), but when they did, it was associated with viewing them positively. Women experienced these interactions at higher levels (see Table 1), but this greater exposure was not significantly associated with their views about street harassment (we return to this point in our discussion section).

Table 3 presents tests of differences in coefficients between Black and Latinx women and men. The tests show that the significant differences in scenario interpretations between White women and men—as seen in Table 2—extended to the other groups. Tests showed the four coefficients for Latinos were significantly larger than those for Latinas, showing Latinos’ greater acceptance of street harassment. The coefficients for Black men were also consistently larger than those for Black women and two of the differences between the coefficients were statistically significant with a two-tailed test (hey sexy and kissing noises scenarios) while a third difference was statistically significant with a one-tailed test (being followed).

**Table 3.** Tests of Differences in Coefficients From Table 2 (*n*  =  163).

Scenario	Comparison groups		*b* ^a^	*b* ^a^	*Z*
Smile	Latino	Latina	−2.00	−0.56	−2.54*
	Black men	Black women	−1.89	−1.17	−1.41
Hey sexy	Latino	Latina	−2.01	−0.18	−3.27***
	Black men	Black women	−2.11	−1.08	−1.97*
Kissing noises	Latino	Latina	−1.54	−0.31	−2.38*
	Black men	Black women	−1.24	−0.24	−2.24*
Being followed	Latino	Latina	−1.48	−0.32	−2.61**
	Black men	Black women	−1.03	−0.36	−1.85

a Unstandardized regression coefficients (see Tables 2 and 3).

***p ≤ .001, **p ≤ .01, *p ≤ .05 (two-tailed).

In Table 4, we introduced our two additional variables: support for sexual street harassment myths *and* prior experience initiating the scenario behaviors. Both variables had notable associations with interpretations and reduced associations between views and the intersection of race/ethnicity and gender. Support for sexual harassment myths was significantly related to interpretations of three of the four scenarios (hey sexy scenario is the exception), and prior experience initiating the behaviors was associated with all four. Introducing these two variables notably reduced many of the relationships evident in Table 2. When comparing Table 2 with Table 4, the significant associations for Black women dropped by more than 40% and only one remained significant (for the smile scenario). Including support for street harassment myths and prior experience instigating also reduced the associations for *all* three groups of men by between 25% and 66%. For White men, two of the associations that were significant in Table 2 were nonsignificant in Table 4; in contrast, the coefficients for Latinos and Black men remained significant. The results indicate that Black, White, and Latinx men's acceptance of street harassment are informed by their support for sexual harassment myths and prior experiences instigating the harassment.^3^

**Table 4.** Ordinary Least Squares (OLS) Regression, Street Harassment, Additional Equations (*n*  =  163).

Variables	Smile^a^	Hey sexy	Kissing noises	Being followed
White men	−0.34	−0.84*	−0.22	−0.55*
	(0.36)	(0.36)	(0.33)	(0.24)
Latinas	−0.43	−0.11	−0.28	−0.30
	(0.35)	(0.30)	(0.29)	(0.28)
Latinos	−1.31***	−1.18**	−0.90*	−1.07**
	(0.39)	(0.42)	(0.38)	(0.34)
Black women	−0.69*	−0.61	0.04	−0.12
	(0.32)	(0.38)	(0.29)	(0.22)
Black men	−1.17**	−1.44***	−0.71*	−0.70*
	(0.39)	(0.36)	(0.34)	(0.29)
Sexual orientation	0.09	−0.07	−0.16	−0.19
	(0.26)	(0.25)	(0.24)	(0.22)
Age	−0.07	−0.12*	−0.05	0.04
	(0.05)	(0.05)	(0.05)	(0.05)
Education	−0.07	0.01	0.06	0.10
	(0.06)	(0.05)	(0.06)	(0.05)
Income	0.07	0.04	0.01	0.04
	(0.04)	(0.04)	(0.05)	(0.04)
Prior target	0.04	−0.25	−0.06	−0.15
(Scenario specific)	(0.12)	(0.13)	(0.14)	(0.10)
Prior initiator	−0.43**	−0.72***	−0.62**	−0.34*
(Scenario specific)	(0.15)	(0.14)	(0.21)	(0.14)
Myth support	−0.22*	−0.18	−0.20*	−0.17*
	(0.09)	(0.10)	(0.08)	(0.07)
Constant	6.85***	7.28***	6.80***	6.23***
	(0.74)	(0.69)	(0.70)	(0.59)

a Unstandardized regression coefficients; robust standard errors in parentheses.

***p ≤ .001, **p ≤ .01, *p ≤ .05 (two-tailed).

## Discussion

More than 25 years ago, Davis (1994, p. 161) asserted, “[b]y refusing to acknowledge difference, street harassment discourse has excluded African American women's experiences.” Since the 1990s, street harassment research has made slow progress on this front. Even more so, it has neglected an intersectional analysis—of at least race and gender—to this harm. This exploratory study answers the call to move beyond a gender-based analysis by investigating how gender *and* race/ethnicity jointly informs perceptions (Davis, 1994). Our study expands the discourse while simultaneously adding complexity to the understanding of street harassment.

Our first finding reports that Black, Latina, and White women perceived the scenarios as impolite, not complimentary, and as harassment relative to Black, Latino, and White men. Our results challenge the claims that Black and Latina women are more tolerant of street harassment because of cultural norms such as “Black cultural scripts” (Kochman, 1981) or linguistic practices manifested through *piropos* (Achugar, 2001). Such results are not surprising as women—particularly women of color—must contend with stereotypes about heterosexual availability and promiscuity in public spaces (Fogg-Davis, 2006). Given that street harassment is a manifestation of sexual terrorism, women must also consider safety in public as they are commonly targeted. Our finding provides an answer to the question of how women interpret harassment in comparison to men in their race/ethnic group: as harassment. Prior work has speculated that this is the case (Davis, 1994; Fogg-Davis, 2006; Gardner, 1995), but ours is the first study, that we know of, to provide quantitative, systematic evidence of it.

Comparisons among Black, Latina, and White women's responses uncovered consistencies and a variance in street harassment views. There were no significant differences between Latinas and White women's interpretations. A possible explanation for such similarities could be the language accessibility for Latinas. Studies on sexual harassment experiences among indigenous and Latina Mexican immigrant farmworkers reported women more readily disclosed harassment experiences when interpretative services were available (Murphy et al., 2016). Fourteen percent of our participants completed the survey in Spanish and removing the language barrier may have made the survey more accessible to Latinas thereby creating a more balanced language field.

Black and White women had similar responses to the kissing noises, being told “hey sexy,” and being followed scenario; they differed with the smile scenario. Such similarities are notable as previous studies described Black women upholding a romanticized interpretation of street harassment (Gardner, 1995) or viewing sexual harassment as less problematic (Welsh et al., 2006) in comparison to White women. The difference between White and Black women in the smile scenario may reflect differences in the contextual nature of street harassment definitions. Whatley's (2018) analysis showed Black women's definition of street harassment focused on explicitly sexual and aggressive behaviors from strangers. It may be that Black women may have considered the smile scenario as less sexually explicit than the other behaviors. Yet, Davis (1994) has previously challenged such a view, arguing that the smile genre of street harassment is a form of domination as it controls women's—particularly Black women's—emotional and intellectual growth.

Historically and currently, the role of smiling is debated beyond the street harassment discourse. As Vera-Gray and Fileborn (2018) assert, smiling is undeservedly cast to the margins of the sexual violence conversation, particularly because it is contrasted to physical forms of violence, such as rape. Our study reinforced this pattern as both women and men across all race/ethnicities marked the physical scenarios (hearing kissing noises and being followed) as more harassing than the verbal interactions (being asked for a smile and being told “hey sexy”). Such results underscore the implicit sexual violence hierarchy whereby physical and/or sexual violence is given a higher positionality than being asked for a smile, as this harm is regarded as an everyday minor annoyance. Within the past few years, the conversation around smiling surfaced in the limelight because of Tatyana Fazlalizadeh's—a Black and Iranian woman—“Stop Telling Women to Smile” campaign. Future studies will need to continue the capture developments and shifts in people's definitions and perceptions of street harassment.

Our study also showed sexual harassment myths and instigating street harassment behaviors were crucial contributors to sexual harassment views, as they came between the relationship between race/ethnicity, gender, and street harassment interpretations. These patterns reinforce DelGreco et al.'s (2020) finding of a significant association between men's tolerance for sexual harassment and their engagement in street harassment. According to DelGreco et al. (2020), men were most likely to engage in street harassment if they believed they had a lower power status than women; future studies should continue to measure levels of perceived power among men of different race/ethnicities.

Our research also highlighted the complex gendered relationships between experiencing street harassment and interpretations. Our analysis did not find a significant association between women's having been targeted and their street harassment interpretations, but did show one for men. For men, previous experiences with the scenarios meant they had more acceptable views of the scenarios. Men's construction of street harassment may reflect their views that these behaviors are an invitation for a heterosexual relationship, as noted in Garnder's (1995) romanticized traditionalist perspective. Similarly, DelGreco et al. (2020) reported that the main motivation men cited for street harassment engagement was affection and a means to express care or liking. Our findings, in addition to DelGreco et al.'s (2020) work, are consistent with the argument that heterosexist masculinity is institutionalized and verified in public settings and allows men to view being asked for a smile or being followed as a romantic invitation.

Echoing other research (Gardner, 1995; Kearl, 2015, 2018; Nilsen, 2000; Raj et al., 2019), we found high levels of street harassment experiences among women: 100% of women in our study said men had told them to smile, 97% said men called them “hey sexy,” 92% said men made kissing noises at them, and 92% said men followed them after calling them “hey baby.” Our study found no significant differences in the frequency of being targeted among Latinas, and Black and White women. Although some earlier studies documented higher rates of street harassment among women of color (Kearl, 2014; Nielsen, 2000; Watson et al., 2015), our findings were consistent with recent research which uncovered comparable rates of street harassment among Latinx, White, Black, and mixed-race women (Raj et al., 2019). Men in our sample reported higher levels of street harassment than in other studies (e.g., Kearl, 2014, 2018): 70% of men said they had been told to smile, 63% said they had been called ‘sexy,’ 53% said kissing noises had been made at them, and 46% said they had been called “hey baby” and being followed.

Street harassment experiences and views interlock with several identities and statuses, such as class (Fileborn & Vera-Gray, 2017), sexual orientation (Fogg-Davis, 2006; Kearl, 2015, 2018), and education. In our study, scenario interpretations were not strongly related to these demographic variables (income, education, etc.). However, there were significant *bivariate correlations* between education, income, and scenario interpretations. Future studies will need to examine further relationships between background variables and street harassment views and experiences.

## Limitations

The study has a small sample, and all participants were surveyed in Northern California. This United States-focused findings may not be generalizable to other countries. Street harassment is a global phenomenon (Kearl, 2015) and its disturbing regularity has been documented in many countries (visit HollaBack.org for more information), resulting in the creation of International Anti-street Harassment Week (see stopstreetharassment.org). Future studies that include an international analysis and application will improve understanding of the complexities, overlapping harms, and commonalities of street harassment. Research should also contextualize harassment within the paternalistic culture and norms of each country.

Our study focused on attitudes toward and experience with verbal types of street harassment (hey sexy and smile scenarios) that integrated physical elements (kissing noises and being followed scenarios). Future survey instruments must examine different forms of street harassment, such as groping or touching (Rosenbaum, 2020). They should also explore more nuanced measures of street harassment victimization, collecting information on how people feel about and interpret their experiences. Subsequent research should also investigate how people view street harassment relative to harassment in other environments, such as the workplace, school, and the internet.

Our study included Spanish and English versions of our survey instrument, but subsequent studies should offer surveys in other languages and use culturally responsive methodologies. Research must move beyond gender binary interactions (meaning, between cis-men and cis-women) and study street harassment in the LGBTQIA+ community (Kearl, 2018; McNeil, 2014) among others with identities closest to the margins. Our study is also limited to Black, Latinx, and White participants and future studies should include the voices of Asians, Native Americans, and others.

Our mediation analysis focused on two variables—street harassment myths and past experiences instigating the harassment—but future studies should interrogate other factors such as feminist identification, toxic masculinity views, and heteronormative beliefs. Future research should explore how these variables mediate, and perhaps moderate, relationships between street harassment views and the intersection of gender, ethnicity, and race.

The principal investigators conducted “safety work” in preparation for the data collection process (Vera-Gray & Kelly, 2020). This type of “safety work” involves preemptive labor to prevent forms of intrusion and escalation from cis-gender men. While the data collectors were under the supervision of the principal investigators during data collection visits, several pedestrians and participants made comments about the data collectors’ appearances and asked for their phone numbers. These interactions extended to one of the principal investigators. In one instance, a White older man completed the survey and then said, “Nice ass!” while pointing to a data collector. This encounter demonstrates how women and feminine-presenting people are vulnerable to street harassment even when they are conducting research on this topic. Future research will need to analyze the experiences of data collectors while collecting data. Studies should also explore how data collectors’ identities and gender expressions influence responses.

Finally, this study calls for an understanding that street harassment is—at the very least—*both gendered and racialized*. Studies must move from a focus on and generalization from White women and intentionally sample racially diverse populations. Investigations must interrogate how women of color perceive their experience as racialized and whether the race of the harasser influences their interpretations; some sexual harassment studies have noted women of color label harassing behaviors differently if the harasser is in their same racial group (Richardson & Taylor, 2009). Researchers should investigate whether this pattern extends to street harassment.

## Conclusion

Street harassment is a manifestation of a sexual imbalance of power connected to patriarchy, racism, and other oppressive forces (Fogg-Davis, 2006). All women are vulnerable to street harassment as it “is a tool of sexual domination available to all men” (Fogg-Davis, 2006, p. 70). A growing literature in the United States has begun to solidify that women and men construct street harassment differently (DelGreco et al., 2020; Gardner, 1995; Kissling, 1991; Nielsen, 2000); however, this is the first study to delineate this pattern by race/ethnicity and explore the role of mediator variables. Our analysis uncovered Black, Latina, and White women were more critical of these behaviors than men in their respective race/ethnic group. Women across all race/ethnicities interpreted the scenarios similarly, with the exception of the smile scenario. Lastly, prior experiences instigating street harassment and support for sexual myths mediated some of the relationships between the intersection of race/ethnicity and gender and scenario interpretations. As the street harassment discourse expands, researchers and activists must strategize intentional and community-responsive methods to combat street harassment outside of police and state interventions. This study advocates for avenues to unlearn and deconstruct heteropatriarchal norms manifested in public spaces that are physically and psychologically detrimental to women.

## Notes

1. We collected data 14 times in an Oakland park, six times in a Sacramento flea market, and once each in a Sacramento park and a Richmond flea market.

2. Survey response rates have declined precipitously (Brick & Williams, 2013). We asked 1,104 people to participate for a response rate of 18%. Response rates of this magnitude are common and not necessarily problematic; indeed, some studies find that low response rates surveys are as accurate as those with higher rates (Keeter et al., 2006). Low response rates are disconcerting when refusal is related to the topic of the study; this was less of a concern for us because most nonrespondents declined to participate before data collectors explained the study.

3. Our data are cross-sectional, however, for most people, experiences and attitudes are mostly acquired after gender and racial/ethnic identities. Seemingly unrelated regression (UCLA, n.d.) offer evidence of mediation, even in small sample studies (Fritz & Mackinnon, 2007). In our analysis, 17 of 24 mediation relationships for men were sizable and statistically significant (results available on request): eight involved support for sexual street harassment myths (four each for Latinos and Black men) and nine involved having prior experience with initiating the behavior in the scenario (four for Black men, three for White men, and two for Latinos).
